# Inhibitory effects of pharmacological doses of melatonin on aromatase activity and expression in rat glioma cells

**DOI:** 10.1038/sj.bjc.6603935

**Published:** 2007-08-14

**Authors:** A González, C Martínez-Campa, M D Mediavilla, C Alonso-González, E J Sánchez-Barceló, S Cos

**Affiliations:** 1 Department of Physiology and Pharmacology, School of Medicine, University of Cantabria, Santander 39011, Spain

**Keywords:** melatonin, pineal, glioma cells, C6 cells, aromatase

## Abstract

Melatonin exerts oncostatic effects on different kinds of neoplasias, especially on oestrogen-dependent tumours. Recently, it has been described that melatonin, on the basis of its antioxidant properties, inhibits the growth of glioma cells. Glioma cells express oestrogen receptors and have the ability to synthesise oestrogens from androgens. In the present study, we demonstrate that pharmacological concentrations of melatonin decreases the growth of C6 glioma cells and reduces the local biosynthesis of oestrogens, through the inhibition of aromatase, the enzyme that catalyses the conversion of androgens into oestrogens. These results are supported by three types of evidence. Firstly, melatonin counteracts the growth stimulatory effects of testosterone on glioma cells, which is dependent on the local synthesis of oestrogens from testosterone. Secondly, we found that melatonin reduces the aromatase activity of C6 cells, measured by the tritiated water release assay. Finally, by (RT)–PCR, we found that melatonin downregulates aromatase mRNA steady-state levels in these glioma cells. We conclude that melatonin inhibits the local production of oestrogens decreasing aromatase activity and expression. By analogy to the implications of aromatase in other forms of oestrogen-sensitive tumours, it is conceivable that the modulation of the aromatase by pharmacological melatonin may play a role in the growth of glioblastomas.

The role of melatonin in tumour development has been under intensive study during the last few decades. It has been reported that melatonin inhibits tumour growth in a variety of *in vivo* and *in vitro* experimental models of neoplasia (Cos and Sánchez-Barceló, 2000a, 2000b; Blask *et al*, 2002). The oncostatic actions of melatonin have been demonstrated at length on hormone-dependent tumours and especially on oestrogen-dependent breast cancer (Sánchez-Barceló *et al*, 2003, 2005). Experimental manipulations activating the pineal gland, or the administration of melatonin, reduce the incidence and growth rate of chemically induced mammary tumours in rodents, while pinealectomy or situations which implicate a reduction of melatonin production usually stimulate mammary carcinogenesis (Cos and Sánchez-Barceló, 2000a, 2000b; Cos *et al*, 2006a). Melatonin reduces the oestrogen-mediated development of breast cancer on the basis of two different mechanisms: indirectly, by the downregulation of the neuroendocrine reproductive axis and the consequent reduction of pituitary and gonadal hormones responsible for the normal and pathological growth of the mammary gland, and, on the other hand, by a direct interaction with the oestrogen-response pathway at the tumour cell level (Cos *et al*, 1998; Sánchez-Barceló *et al*, 2005). It has been demonstrated that melatonin counteracts the effects of oestrogens on mammary tumoral cells, thus behaving as a selective oestrogen receptor modulator (SERM). Furthermore, melatonin regulates the expression and activity of the aromatase, the main enzyme responsible for the local synthesis of oestrogens, thus behaving as a selective oestrogen enzyme modulator (SEEM) (Cos *et al*, 1991, 2006a; Martínez-Campa *et al*, 2005).

Oestrogens have well-known cytoprotective effects in neural cells and it has been reported to also have biological effects on glial cells, although these have been less studied (Shy *et al*, 2000; Sur *et al*, 2003; Haghighat *et al*, 2004; Mhyre *et al*, 2006; Sribnick *et al*, 2006). Glioblastoma is the most frequent primary malignant CNS tumour in adults. These tumours express oestrogen receptor and also express aromatase (Jung-Testas *et al*, 1992, 1994; Yague *et al*, 2004). Expression of aromatase in the brain is involved in the regulatory effects of androgens, via its conversion into oestrogens, on neural differentiation, maturation, neural plasticity, neuroendocrine functions and sexual behaviour (Lephart, 1996). There is some evidence that tamoxifen, one of the oestrogen receptor modulators most frequently used for treatment and also for prevention of breast cancer, arrests the growth of some glioblastomas and it has neuroprotective actions (Shy *et al*, 2000). Proliferative signal transduction in glioma cells occurs predominantly through a protein kinases C-dependent pathway, and tamoxifen has been demonstrated to be effective in blocking cell growth in glioma cell lines by inhibiting these protein kinases C (Baltuch and Yong, 1996; Luo *et al*, 1997; Bergan *et al*, 1999).

Melatonin has been shown to have neuroprotective effects in a large number of different experimental models (Cos *et al*, 1996; Armstrong and Niles, 2002; Feng and Zhang, 2004; Rincón Castro *et al*, 2005; Martín *et al*, 2006; Herrera *et al*, 2007). However, there is limited evidence suggesting that melatonin may affect glioblastoma growth. In a clinical trial in which melatonin was administered to patients being treated with radiation therapy for glioblastoma, a significant increase in 1-year survival was seen (Lissoni *et al*, 1996). C6 glioma cells express both of the protein-coupled melatonin receptor subtypes, MT_1_ and MT_2_ and when they are treated with physiological or higher concentrations of melatonin, a significant induction of relative glial cell line-derived neurotrophic factor has been detected (Armstrong and Niles, 2002). This finding has suggested that induction of the glial cell line-derived neurotrophic factor is involved in physiological neuroprotection by melatonin. High doses of valproic acid, a potent antiepileptic, induce both upregulation of melatonin receptors and expression of neurotrophic factors in C6 glioma cells (Rincón Castro *et al*, 2005). Recently, it has been reported that melatonin at millimolar concentrations reduces, both *in vitro* and *in vivo*, the growth of C6 glioma cells (Martín *et al*, 2006). This inhibition has been associated with a decrease in basal levels of intracellular free radicals. In addition to the beneficial effects of providing direct antioxidant protection to glioma cells, melatonin may enhance neuroprotection against A*β*-induced neurotoxicity by attenuating A*β*25-35 or A*β*1-42-induced apoptosis and promoting the survival of glial cells (Feng and Zhang, 2004).

Although C6 glioma cells express both oestrogen receptor and aromatase and have, therefore, the possibility to produce oestrogens from androgen precursors, which may act as paracrine or autocrine factors to promote tumour cell growth, the significance of this fact is not very well determined. In other oestrogen-dependent tumours, such as mammary tumours, the inhibition of aromatase activity by aromatase inhibitors is currently one of the first therapeutic strategies used against the growth of these tumours (Kitawaki *et al*, 1992; Burak *et al*, 1997). Since melatonin inhibits the growth of C6 cells, and this indoleamine has been demonstrated to be able to decrease aromatase expression and activity in other tumour cell lines, our objective in the present study was to analyse the possible effects of melatonin on the local synthesis of oestrogens in C6 glioma cells and its influence on the cell proliferation.

## MATERIALS AND METHODS

### Chemicals

Unless otherwise indicated, all chemicals used in this work were purchased from Sigma-Aldrich (Madrid, Spain).

### Cells and culture conditions

Rat glioma cells (C6) were a kind gift from Dr Carmen Rodríguez from the Department of Structural and Cellular Biology of the University of Oviedo (Spain). They were maintained as monolayer cultures in 75 cm^2^ plastic culture flasks in Dulbecco's modified Eagle's medium (DMEM)/HAM's nutrient mixture F-12 supplemented with 10% fetal bovine serum (FBS) (PAA Laboratories GmdH, Pusching, Austria), 20 U ml^−1^ penicillin and 20 *μ*g ml^−1^ streptomycin, at 37°C in a humid atmosphere containing 5% CO_2_. Cells were subcultured every 3–4 days by suspension in 5 mM Na_2_-EDTA in PBS (pH 7.4) at 37°C for 5 min.

Before each experiment, stock subconfluent monolayers (80%) of glioma cells were incubated with 5 mM Na_2_-EDTA in PBS (pH 7.4) at 37°C for 5 min, re-suspended in DMEM/HAM F12 mixture supplemented with 10% FBS and passed repeatedly through a 25-gauge needle to produce a single cell suspension. Cell number and viability were determined by staining a small volume of cell suspension with 0.4% Trypan blue saline solution and examining the cells in a haemocytometer.

### Measurement of cellular proliferation

Glioma cells were seeded in 96-multiwell plates at a density of 4000 cells per well, in DMEM/HAM F12 mixture with 10% FBS, 20 U ml^−1^ penicillin and 20 *μ*g ml^−1^ streptomycin, at 37°C in a humid atmosphere containing 5% CO_2_. After 24 h of incubation to allow a correct attachment of the cells, media were renewed containing either 1 mM, 10 *μ*M, 1 *μ*M, 100 nM, 1 nM, 10 pM melatonin and/or 10 nM oestradiol and/or the diluent of these drugs (ethanol, at final concentration lower than 0.0001% per plate). Cells were cultured for 3 or 4 days. Cell proliferation was measured by the MTT method reading absorbance at 570 nm in a microplate reader.

### Indirect measurement of aromatase activity

Indirect evidence of aromatase activity of C6 glioma cells was obtained by evaluating cell proliferation in oestrogen-free media in the presence of testosterone. C6 glioma cells were seeded into 96-well culture plates at a density of 4000 cells per well, in DMEM/HAM F12 mixture supplemented with 10% FBS, 20 U ml^−1^ penicillin 20 *μ*g ml^−1^ streptomycin, at 37°C in a humid atmosphere containing 5% CO_2_. After 24 h of incubation, media were changed to others supplemented with 5% charcoal-stripped FBS (sFBS) containing either 1 *μ*M testosterone, 1 mM melatonin, 100 *μ*M aminoglutethimide (an aromatase inhibitor), or the diluent of these drugs (ethanol, at final concentration lower than 0.0001% per plate). Cell proliferation was assessed at 3 and 4 days of culture, by using the MTT method, reading absorbance at 570 nm in a microplate reader.

### Direct measurement of cellular aromatase activity

Aromatase activity in C6 glioma cells was measured by the tritiated water release assay, based on the formation of tritiated water during aromatisation of an androgenic substrate such as [1*β*-^3^H(N)]-androst-4-ene-3,17-dione (Ackerman *et al*, 1981). Glioma cells were seeded onto 60 × 15 mm tissue culture dishes (2 × 10^6^ cells per dish) in DMEM/HAM F12 mixture supplemented with 10% FBS, 20 U ml^−1^ penicillin and 20 *μ*g ml^−1^ streptomycin. After 24 h, when a homogeneous monolayer of preconfluent C6 cells was reached, media were aspirated and replaced by fresh media (1 ml per plate) supplemented with 5% sFBS and containing 100 nM [1*β*-^3^H(N)]-androst-4-ene-3,17-dione] (NEN Life Science Products, Boston, MA, USA) (25–30 Ci mM^−1^) in the presence of 1 mM, 1 *μ*M or 1 nM melatonin or its diluent (ethanol at a final concentration lower than 0.0001%). At 24 h of incubation, the culture dishes were placed on ice for 15 min to condense any water vapour and the media were transferred to tubes containing 0.25 ml ice-cold 30% trichloroacetic acid (wt vol^−1^), vortexed and centrifuged at 1700 **g** for 15 min. The supernatants were extracted with chloroform, vortexed, set at room temperature for 10 min and then centrifuged at 1700 **g** for 15 min. The resulting aqueous supernatants were adsorbed with 10% dextran-coated charcoal, vortexed, centrifuged at 1700 **g** for 15 min and the supernatant added to vials with scintillation cocktail and counted in a beta counter. The amount of radioactivity in water [^3^H] measured was corrected by subtracting the blank values from each sample, obtained by incubating dishes containing medium with the tritiated androgen but no cells. The values were also corrected by taking into account the fractional retention of tritium in medium water throughout the procedure of incubation and processing, utilising parallel dishes containing medium plus known amounts of [^3^H] water (NEN Life Science Products) through incubation and assay. The fractional retention of tritium in medium water throughout the incubation and processing of samples was always higher than 85%.

### Measurement of aromatase mRNA expression

Analysis of the aromatase mRNA expression in C6 glioma cells was carried out by real-time reverse transcription (RT)–PCR. The total cellular RNA was purified with the Nucleospin RNA II Kit (Macherey-Nagel, Düren, Germany) following the manufacturer's instructions. Integrity of RNA was assessed by electrophoresis in ethidium bromide-stained 1.2% agarose-Tris-borate EDTA gels. The absorbance ratio A_260 nm_/A_280 nm_ was greater than 1.8. For cDNA synthesis, 1 *μ*g of total RNA was denaturated at 65°C for 10 min and reverse-transcribed 50 min at 45°C with cDNA Synthesis kit (Bioline, London, UK) in a final volume of 20 *μ*l in the presence of 500 ng of oligo (dT)12–18 primer.

PCR was performed using a set of rat aromatase-specific primers (5′–TATTGGAAATGCTGATTGCGG (forward) and 5′-TTGGGCTTGGGGAAATACTCG (reverse)) (Sigma Genosys Ltd, Cambridge, UK). The coding sequence between the two PCR primer sites is interrupted by three introns in the gene. As a control quantification, S14 mRNA was also carried out by RT–PCR using a set of specific primers (5′-TCACCGCCCTACACATCAAAC (forward) and 5′-TCCTGCGAGTGCTGTCAGAG (reverse)) (Sigma Genosys Ltd).

Real-time PCRs were performed in a MX3000 (Stratagene, La Jolla, CA, USA) using Brilliant® SYBR® Green PCR Master Mix (Stratagene) following the manufacturer's instructions. Amplifications were performed for 40 cycles using the following temperature profile: 55°C, 60 s (annealing); 72°C, 30 s (extension); and 95°C, 30 s (denaturation).

### Statistics

The data on cell proliferation or aromatase activity and expression are expressed as the mean±s.e.m. Statistical differences between groups were processed by one-way analysis of variance (ANOVA) followed by the Student–Newman–Keuls test.

## RESULTS

**Figure 1:**
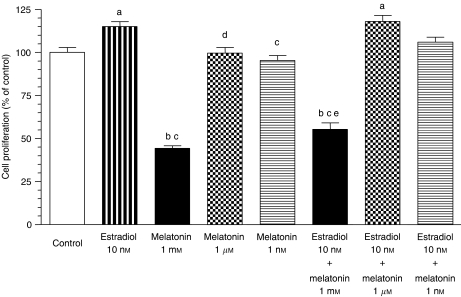
Effects of melatonin (1 mM, 1 *μ*M and 1 nM) and oestradiol (10 nM), either alone or in combination, on C6 glioma cells proliferation. Cells were seeded into 96-well culture plates (4000 cells per well) in medium supplemented with 10% fetal bovine serum (FBS) for 24 h. Then the media were aspirated and replaced by fresh media supplemented with 5% charcoal-stripped FBS (sFBS) and containing the indicated concentrations of oestradiol and/or melatonin. After 4 days, cell proliferation was measured by the MTT method. Data are expressed as the percentage of the control group (mean±s.e.m.). a, *P*<0.05 *vs* control; b, *P*<0.001 *vs* control; c, *P*<0.001 *vs* oestradiol 10 nM; d, *P*<0.05 *vs* oestradiol 10 nM; e, *P*<0.05 *vs* melatonin 1 mM.

Incubation with 17*β*-oestradiol (10 nM) significantly stimulated the proliferation of C6 cells (Figure 1). The simultaneous addition of 17*β*-oestradiol and melatonin resulted in a significantly (*P*<0.001) lower cell proliferation than that of the 17*β*-oestradiol-treated cells and control (untreated) cells. Melatonin 1 *μ*M and 1 nM were not able to counteract the stimulatory effects of 17*β*-oestradiol (Figure 1).

**Figure 2:**
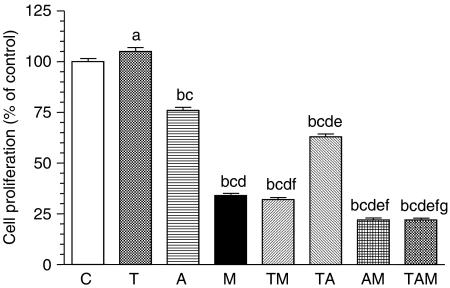
Indirect measurement of aromatase activity. Effects of 1 *μ*M testosterone (T), 1 mM melatonin (M), 100 *μ*M aminoglutethimide (A), or the diluent of these drugs (ethanol 0.0001%) (C), either alone or in combination on glioma cell proliferation. Cells were seeded into 96-well culture plates (4000 cells per well) in medium supplemented with fetal bovine serum (FBS) for 24 h and subsequently for 4 days in medium supplemented with charcoal-stripped FBS (sFBS) containing the above-mentioned drugs. Data are expressed as the percentage of the control group (mean±s.e.m.). a, *P*<0.01 *vs* C; b, *P*<0.001 *vs* C; c, *P*<0.001 *vs* T; d, *P*<0.001 *vs* A; e, *P*<0.001 *vs* M; f, *P*<0.001 *vs* TA; g, *P*<0.001 *vs* TM.

Indirect evidence of aromatase activity of glioma cells was obtained by evaluating cell proliferation in oestrogen-free media in the presence of testosterone. Testosterone (10 *μ*M) significantly increased proliferation of C6 cells cultured for 4 days in media with sFBS (Figure 2). This stimulatory effect was reduced (*P*<0.001) by the aromatase inhibitor aminoglutethimide, thus indicating that, at least in part, cell proliferation was dependent on the formation of oestrogens from testosterone by the aromatase activity of the cells. Melatonin (1 mM) was also able to counteract the stimulatory effect of testosterone to values below those obtained with aminoglutethimine, thus suggesting that it also exerts inhibitory effects on aromatase (Figure 2).

**Figure 3:**
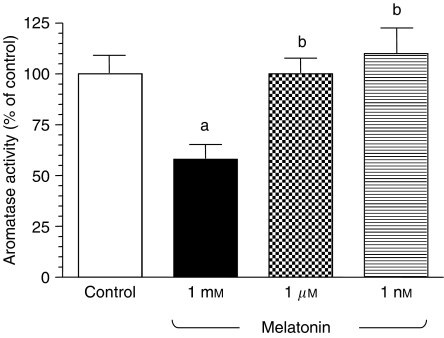
Effects of melatonin (1 mM, 1 *μ*M or 1 nM) or the diluent (ethanol at 0.0001% final concentration) on aromatase activity of glioma cells. Cells were seeded onto 60 × 15 mm dishes (2 × 10^6^ cells per dish) in Dulbecco's modified Eagle's medium (DMEM)/HAM F12 mixture supplemented with 5% FBS for 24 h. Then, media were aspirated and replaced by fresh media supplemented with 5% charcoal-stripped FBS (sFBS) and containing tritiated androstenedione and the indicated concentrations of melatonin. Aromatase activity was determined after 24 h of incubation, as described in Materials and Methods. Data are expressed as the percentage of the control group (mean±s.e.m.). a, *P*<0.05 *vs* control; b, *P*<0.05 *vs* melatonin 1 mM.

The aromatase activity of C6 glioma cells incubated for 24 h with tritiated androstenedione was estimated by the formation of tritiated water. Figure 3 shows that only melatonin 1 mM significantly decreases aromatase activity of C6 cells. Melatonin 1 mM induced a significant 40% inhibition of the aromatase activity of glioma cells.

**Figure 4:**
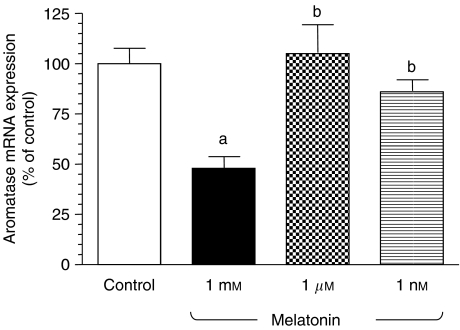
Effects of melatonin on the expression of aromatase mRNA in glioma cells. Cells were incubated with melatonin (1 mM, 1 *μ*M or 1 nM) or ethanol (0.0001%; control) for 90 min. Total mRNA was isolated from C6 cells and reverse transcribed. cDNA was subjected to PCR using specific primers for *P*450 aromatase or S14. Data are expressed as the percentage of the control group (mean±s.e.m.). a, *P*<0.01 *vs* control; b, *P*<0.05 *vs* melatonin 1 mM.

To determine whether this inhibitory effect of melatonin over aromatase activity was due to a downregulation of the aromatase expression at the transcriptional level, we then incubated glioma cells with either melatonin (1 mM, 1 *μ*M or 1 nM) or vehicle for 90 min and total RNA was isolated to perform real-time PCRs with specific primers for rat aromatase. As a control, the same samples were subjected to real-time PCR with specific primers for S14, a ribosomal protein component of the 40S subunit. Melatonin treatment significantly inhibited (*P*<0.01) aromatase mRNA expression in glioma cells at 1 mM concentration (Figure 4).

## DISCUSSION

Sex hormones play a multifaceted role throughout the body. Sex differences in brain tumour incidence suggest that hormonal factors may play a role in the aetiology of this type of tumour and there is evidence that hormones related to female reproductive function may be associated with brain cancer risk (Huang *et al*, 2004). The incidence of glioma is 1.5 times greater in men than in women and it has been described an inverse relationship between younger age at menarche (among postmenopausal women), cumulative number of menstrual cycles over the lifetime, fewer months of breast-feeding and the use of hormone-replacement therapy and the risk for glioma (Inskip *et al*, 1995; Huang *et al*, 2004). Steroids or their precursors can be metabolised in the CNS to derivates which can affect brain function and may have an important clinical significance (Weidenfeld and Schiller, 1984). Previous studies have shown that human brain and human neuronal cell lines secrete oestrogens (Stoffel-Wagner, 2001, 2003).

Melatonin, the main pineal hormone, inhibits the growth of C6 rat glioma cells both *in vitro* and *in vivo*, and this inhibition has been related with the antioxidant abilities of this indolamine (Martín *et al*, 2006). In other models of oestrogen-sensitive tumours, such as breast cancer, melatonin oncostatic actions seem to be mainly based on its interaction with the tumour cells'oestrogen-response pathway (Cos and Sánchez-Barceló, 2000a, 2000b; Del Rio *et al*, 2004; Cos *et al*, 2006a). Melatonin interacts with oestradiol at the oestrogen receptors level in the mammary tumour cell and also regulates the expression and the activity of the aromatase responsible for the local synthesis of oestrogens. Thus, a single molecule has both SERM and SEEM properties, one of the main objectives desired for the breast antitumour drugs (Cos *et al*, 2005, 2006a, 2006b; Martínez-Campa *et al*, 2005). For that reason, we decided to address whether the modulation of the local biosynthesis of oestrogens could be involved in the inhibition of C6 glioma cells by melatonin.

According to previous findings (Martín *et al*, 2006), only 1 mM melatonin added to the culture decreased C6 cell growth. This melatonin concentration reduced C6 growth by 30% after 4 days of culture. In the same way, also high concentrations of melatonin are also necessary to obtain oncostatic actions in neuroblastoma cells and other kinds of tumour cells (Sainz *et al*, 2005; García-Santos *et al*, 2006). Melatonin is a highly lipid-soluble indolamine which may easily cross the blood–brain barrier, and there is evidence that melatonin concentration in the cerebrospinal fluid is higher than in blood (Rousseau *et al*, 1999; Longatti *et al*, 2007). These high concentrations of melatonin in the cerebrospinal fluid may contribute to explain why high levels of melatonin are necessary to inhibit glioblastoma cell proliferation.

Oestrogen receptors present in primary glial cell cultures and different types of glial cells (Jung-Testas *et al*, 1992; Santagati *et al*, 1994; Yague *et al*, 2004) allow this non-neuronal cells of the CNS to respond to oestrogens; so glioma cells were able to respond significantly to oestradiol in the culture. Melatonin, only at 1 mM concentration, counteracted the growth stimulatory effect of oestradiol on C6 cells. In addition, previous studies have shown that the human brain, human neuronal cell lines and glioma cell lines express aromatase and, are, therefore, capable of producing oestrogens from androgens (Stoffel-Wagner, 2001; Yague *et al*, 2004).

The present study demonstrates that melatonin, at 1 mM concentration, reduces the synthesis of oestrogens in C6 glioma cells, through the inhibition of aromatase, the enzyme that catalyses the rate-limiting step in the conversion of androgens into oestrogens (Zhou *et al*, 1993). These results are supported by the three types of experiments we carried out. In the first experimental series, we demonstrate that melatonin counteracts the growth stimulatory effects of testosterone on C6 glioma cells (Merritt and Foran, 2007) cultured in oestrogen-free media. Under these conditions, cell growth depends on the biotransformation of androgens to oestrogens via their own aromatase activity. Consequently, the inhibitory effects of melatonin could be explained because of its antiaromatase activity. In a second series of experiments, we directly quantified the aromatase activity of glioma cells, finding that this was reduced by melatonin at 1 mM concentration, the same concentration that causes the greatest antiproliferative effects in these cells (Martín *et al*, 2006). Finally, we demonstrated that 1 mM melatonin induced a remarkable decrease in the expression of mRNA aromatase of C6 glioma cells.

To our knowledge this is the first time that the influence of melatonin on aromatase expression and activity of glioblastoma cells has been established. In fact, the antiaromatase activity of melatonin in other cells has not been firmly established until recently. A limited number of earlier studies had brought controversial results. Thus, no consistent effects of melatonin on aromatase activity were described in human, porcine or bovine granulose cells cultured in a serum-supplemented medium (Webley and Luck, 1986; Tanavde and Maitra, 2003). Other authors attributed the low sperm quality of human seminal plasma to a low aromatase activity dependent on high endogenous melatonin levels (Yie *et al*, 1991) or long-term melatonin administration (Luboshitzky *et al*, 2002). A reduction in the aromatase activity in the hypothalamic–preoptic area of adult male Syrian hamsters chronically treated with melatonin (Petterborg *et al*, 1991) has also been described, but considered secondary to the decreased circulating levels of testosterone. It was our group that recently described, in the first instance, that melatonin works as a SEEM in breast cancer cells (*in vitro*) as well as in DMBA-induced rat mammary tumours (*in vivo*) (Cos *et al*, 2005, 2006b).

Our results indicate that oestrogens locally produced by aromatase in glioblastoma cells may act as autocrine or paracrine factors and melatonin is able to inhibit this local production of oestrogens by decreasing aromatase activity and expression. The significance of this melatonin action on aromatase in glioblastoma needs to be determined. There is little information about the capacity of C6 glioma cells to growth in the presence of aromatase inhibitors; however, by analogy to the implications of aromatase in other forms of oestrogen-sensitive tumours, such as breast tumours, it is conceivable that the modulation of the activity and expression of aromatase by pharmacological concentrations of melatonin may play a role in the growth of glioblastomas.
